# Identification of the active components in Bone Marrow Soup: a mitigator against irradiation-injury to salivary glands

**DOI:** 10.1038/srep16017

**Published:** 2015-11-03

**Authors:** Dongdong Fang, Shen Hu, Younan Liu, Vu-Hung Quan, Jan Seuntjens, Simon D. Tran

**Affiliations:** 1 Craniofacial Tissue Engineering and Stem Cells Laboratory, Faculty of Dentistry, McGill University, Montreal, QC, H3A 0C7, Canada; 2 School of Dentistry, University of California, Los Angeles, CA, 90095, USA; 3 Department of Cardiology, Université de Montréal, Montreal, QC, H2W 1T8, Canada; 4 Department of Oncology, Medical Physics Unit, McGill University, Montreal, QC, H3G 1A4, Canada

## Abstract

In separate studies, an extract of soluble intracellular contents from whole bone marrow cells, named “Bone Marrow (BM) Soup”, was reported to either improve cardiac or salivary functions post-myocardial infarction or irradiation (IR), respectively. However, the active components in BM Soup are unknown. To demonstrate that proteins were the active ingredients, we devised a method using proteinase K followed by heating to deactivate proteins and for safe injections into mice. BM Soup and “deactivated BM Soup” were injected into mice that had their salivary glands injured with 15Gy IR. Control mice received either injections of saline or were not IR. Results at week 8 post-IR showed the ‘deactivated BM Soup’ was no better than injections of saline, while injections of native BM Soup restored saliva flow, protected salivary cells and blood vessels from IR-damage. Protein arrays detected several angiogenesis-related factors (CD26, FGF, HGF, MMP-8, MMP-9, OPN, PF4, SDF-1) and cytokines (IL-1ra, IL-16) in BM Soup. In conclusion, the native proteins (but not the nucleic acids, lipids or carbohydrates) were the therapeutic ingredients in BM Soup for functional salivary restoration following IR. This molecular therapy approach has clinical potential because it is theoretically less tumorigenic and immunogenic than cell therapies.

During the past decade, the number of patients newly diagnosed with head and neck cancer has doubled to more than 40,000 in the United States^1^,^2^. Surgery and irradiation (IR) remain the most frequently used treatments for head and neck cancer. Despite an improved patients’ survival rate, IR causes inevitably severe side effects, due to the high dose of co-irradiation to normal tissue surrounding the tumor, among which salivary hypofunction (dry mouth, xerostomia) is the most prominent sequela experienced by more than 60% of patients receiving IR for head and neck cancer^3^,^4^. To reduce IR damage to the salivary glands (SG), new radiation techniques such as intensity-modulated radiation therapy with image guidance (IMRT/IGRT) and proton radiotherapy are being used^5^,^6^. Submandibular gland transfer is also an alternative method to spare SG from high dose co-irradiation^7^. However, ~40% of patients receiving IMRT are still suffering from reduced salivary flow^8^, and gland transfer cannot be applied to every patient. Salivary hypofunction (i.e., reduced saliva flow) predisposes patients to morbid conditions such as oral mucositis and infections, dental caries, difficultly speaking, chewing and swallowing food and leading to a diminished quality of life and malnutrition^9^. Unfortunately, current treatments for salivary hypofunction remain palliative and thus radical treatment strategies are required to restore SG function. Gene therapy, tissue engineering and stem cells are currently the three major experimental approaches tested for functional restoration of damaged SG.

Adult stem cell-based therapy has been reported to slow down the apoptotic activity^10^, to normalize the stem/progenitor cell pool^9^, and to improve the function of SG. Several types of stem cells, such as bone marrow (BM)^10^,^11^, adipose-derived stromal cells^12^, dental pulp cells^13^ and SG stem/progenitor cells^9^, were reported to restore function of SG damaged by IR. Initially, the mechanisms of (therapeutic) action proposed that stem cells differentiated into or fused with the tissue parenchymal cells^14^,^15^. The currently proposed mechanism is that transplanted cells (e.g., BM or MSC) secrete paracrine factors, such as cytokines and growth factors, needed for the tissue repair and regeneration process^16^,^17^. Yeghiazarians and colleagues elegantly demonstrated that injection of intact BM cells versus a cell lysate (bone marrow cell extract) resulted in comparable benefits in a mouse model of acute myocardial infarction^18^. However when the whole BM was fractionated into subpopulations of cells (‘fractionated BM cell extract’), then the cardiac functional efficacy was less than that of ‘whole BM cell extract’^19^. These results suggested that numerous factors secreted/released by numerous BM cell populations were responsible for cardiac functional improvement. Interestingly, the same group of researchers injected ‘human’ BM cell extract into the same mouse model and reported improved cardiac function without immune rejection^20^. Thus this BM cell extract is less immunogenic than transplanting whole BM cells. Our group adapted this method and coined the term as “Bone Marrow Soup” (*BM Soup*), which represented all the yet-to-be-identified soluble components of the cell lysate from whole bone marrow cells. We reported that both whole BM cells and BM Soup restored comparable salivary function following IR and in Sjögren’s-like disease mice^21^,^22^. Because the BM Soup is an extract from a cell lysate, it is theoretically less immunogenic and tumorigenic^23^. However, the components which are responsible for these promising therapeutic actions remain unknown.

In this study, we have demonstrated that the protein components but not the nucleic acids, lipids, or carbohydrates in the BM Soup are the active/therapeutic ingredients for functional salivary restoration following IR. We have also employed a protein microarray approach to preliminarily identify important cytokines and growth factors that are present in the BM Soup.

## Results

### Proteins are the active ingredients in BM Soup

**Figure 1:**
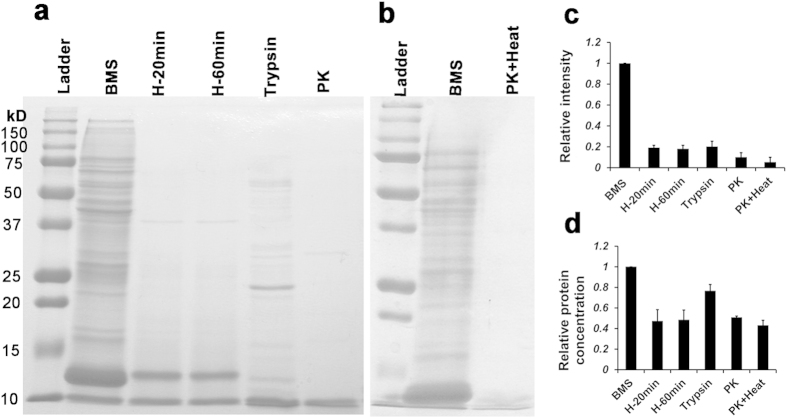
Deactivation of BM Soup. (**a**) Four methods to deactivate the native BM Soup (BMS) were tested, 1) BMS heated at 95 °C for 20 min (H-20 min); 2) heated at 95 °C for 60 min (H-60 min); 3) digested by trypsin (1:20 w/w) at 37 °C for overnight; 4) digested by 0.1 μg/μl Proteinase K (PK) at 37 °C for overnight. (**b**) Proteinase K digestion at 37 °C overnight was followed by heating at 95 °C for 2 min (PK + Heat) to reduce/remove the toxicity of proteinase K when injected into live animals. Protein bands were shown by SDS-PAGE and Coomassie blue staining. (**c**) Relative quantification of proteins (intensity) on the SDS gel was analyzed with NIH Image J software. (**d**) The total protein concentration was measured by the bicinchoninic acid assay method (BCA). Three independent experiments were done. All data were normalized to BM Soup.

Four methods to deactivate proteins were tested in this study: 1) 95 °C for 20 min, 2) 95 °C for 1 hour, 3) trypsin digestion at 37 °C for overnight, and 4) proteinase K at 37 °C for overnight. Coomassie blue staining was used to visualize the proteins after deactivation (Fig. 1a,b). All four methods cleaved/degraded the majority of proteins above 12 kDa. Coomassie staining indicated that the total density for remaining proteins after deactivation from all four methods was 80~90% less intense than that of the native BM Soup (Fig. 1c). Measurements of protein concentration (BCA method) showed a reduction ~20% with trypsin and ~50% with heat or proteinase K alone (Fig. 1d). The proteinase K method resulted in the least amount of proteins observed on the gel and with the smallest peptides. Thus we chose proteinase K (0.1 μg/μl) as the most efficient method to degrade/deactivate proteins from BM Soup. However, considering the toxicity of proteinase K if injected into live animals, an additional step of heating at 95 °C for 20 min was conducted to inactivate proteinase K (Fig. 1b). Almost no protein bands were observed on the gel with Coomassie blue staining.

### *In vivo* injection of ‘Deactivated BM Soup’ does not restore function in irradiated salivary glands to normal levels.

**Figure 2:**
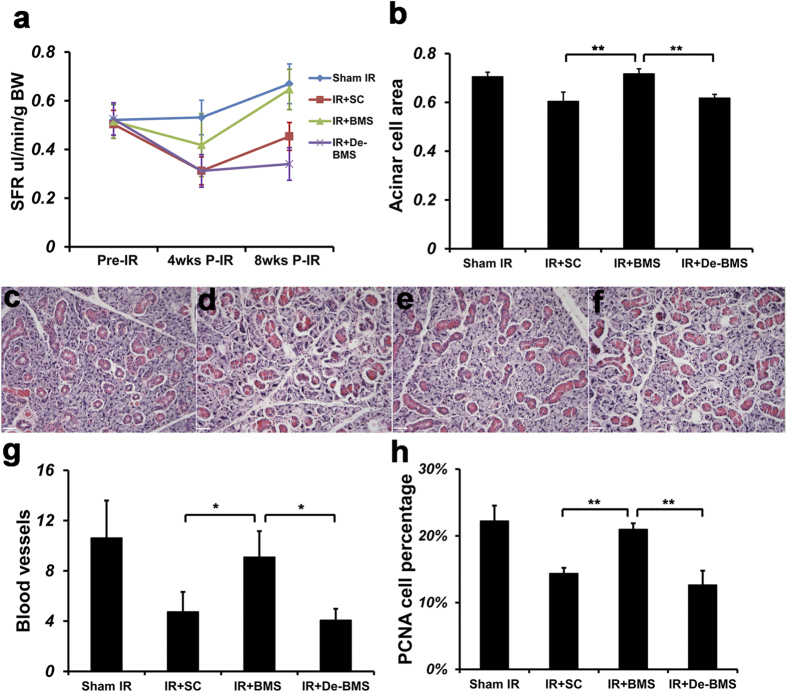
Role of BM Soup and ‘deactivated BM Soup’ on SFR, cell proliferation, acinar cells and blood vessels. (**a**) Salivary flow rate (SFR) was determined by volume of saliva/10min/g body weight at pre-irradiation (Pre-IR), 4 weeks post-IR and 8 weeks post-IR. Then mice were sacrificed and submandibular glands were harvested at 8 weeks post-IR. Histological changes were examined for (**b**) Percentage of tissue surface area occupied by acinar cells/total area; H&E staining of mouse submandibular glands for the (**c**) Sham IR group; (**d**) Saline Control group; (**e**) BM Soup group; (**f**) Deactivated BM Soup group. Scale bar is 38 μm. (**g**) Number of blood vessels were calculated under 200 X magnification of 10 fields per gland/mouse with NIH image J software. (**h**) Percentage of PCNA positive cells was counted under 400 X magnification. Two examiners independently analyzed in a blind manner. *P < 0.05; **P < 0.01, n = 5 mice per group. All data were presented with mean ± S.D. BMS: BM Soup; De-BMS: deactivated BM Soup; SC: saline control.

Salivary flow rate (SFR) is an objective measure of SG function. Results at week 8 post-IR showed that SFR of irradiated mice injected with the vehicle control (saline control; IR + SC group) had a significantly reduced SFR (*P* < 0.05, n = 5) when compared with sham-IR mice (i.e. the negative control group; mice were sedated and placed inside the irradiator but the machine was off; Fig. 2a). SFR of irradiated mice injected with the ‘Deactivated BM Soup’ was comparable to that of irradiated mice injected with saline control (IR + SC group). This meant that the ‘Deactivated BM Soup’ was no better than injections of normal saline in IR mice. SFR of mice in the BM Soup-treated group was comparable to that of sham-IR mice. These data indicated that BM Soup treatment, not the deactivated BM Soup, restored saliva secretory function (Fig. 2a, *P* < 0.05, n = 5). Because the ‘Deactivated BM Soup’ had its active proteins cleaved by proteinase K (while nucleic acids, lipids and carbohydrates were not), these results suggested that the proteins were potential therapeutic ingredients in BM Soup.

In addition to monitoring SFR, we also compared the ‘native BM Soup’ and its deactivated form at the histology, gene, and protein expression levels in irradiated SG. All these measures confirmed the same trend observed with SFR. That is, ‘deactivated BM Soup’ was not better than injection of saline while the native BM Soup protected salivary cells and blood vessels from IR damage, and up-regulated the expression of genes related to SG development and regeneration. Thus the loss of therapeutic effect of the BM Soup was due to the removal (deactivation) of its native protein components.

**Figure 3:**
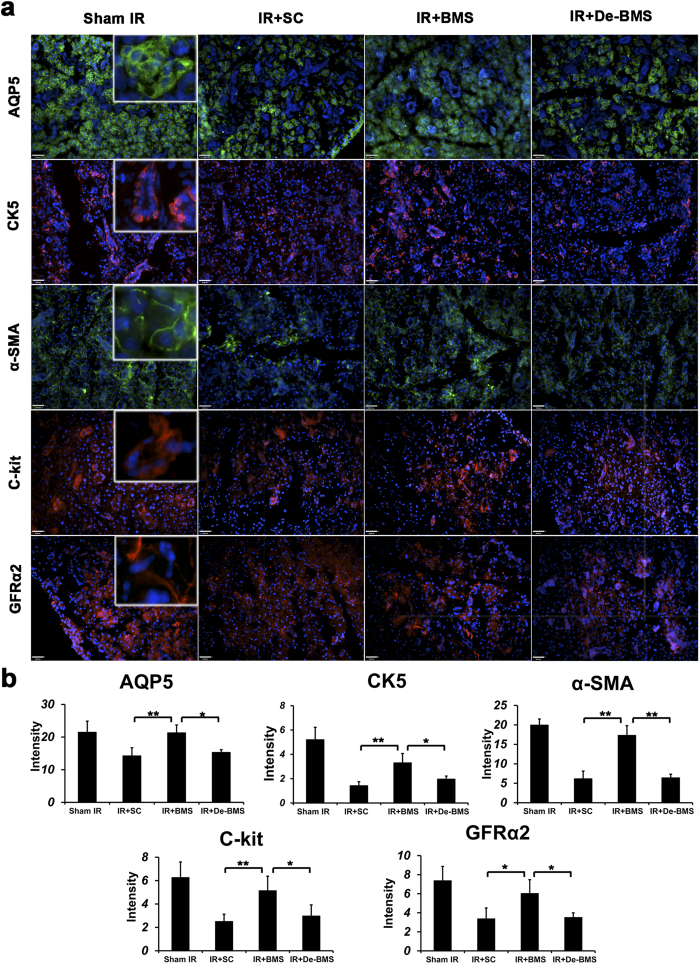
Special subpopulation of gland cells was detected by immunofluorescent staining at 8 weeks post-irradiation. (**a**) The cells positive for AQP5 (marker of acinar cells), α-smooth muscle actin (marker for myoepithelial cells), CK5 (marker for ductal cells), c-Kit (marker for stem/progenitor cells), and GFRα2 (marker for parasympathetic nerves) were detected on frozen sections of salivary glands. Scale bar is 38 μm. All photographs were taken at 200 X magnification. (**b**) Quantification of protein immunofluorescent expression in 5 random fields/glands by Image J software (n = 5 mice per group; *P < 0.05, **P < 0.01). BM Soup group has higher protein intensity, when compared with deactivated BM Soup group. SC: saline control; BMS: BM Soup; De-BMS: deactivated BM Soup.

**Figure 4:**
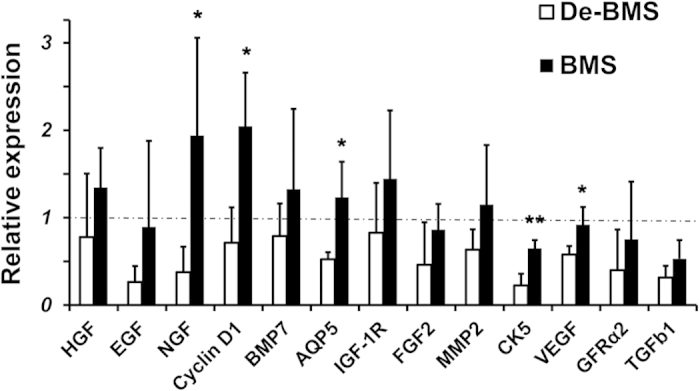
Relative expression of genes in salivary glands at week 8 post-irradiation. Expression levels of genes related to tissue repair/regeneration were analyzed by quantitative RT-PCR. Gene expression levels were significantly higher in BM soup-treated group than that of the deactivated BM soup for AQP5, CK5, VEGF, NGF and Cyclin D1 (cell cycle G1/Synthesis). Y-axis shows the relative expression of the gene compared to GAPDH. Horizontal dashed line represents the relative gene expression level of 1 in mice from the Control group (sham-irradiated mice). *P < 0.05, **P < 0.01; *n* = 5 mice. Three experimental replicates were conducted for each sample. BMS: BM Soup; De-BMS: deactivated BM Soup.

The percentage of surface acinar area/total area was comparable between sham-IR and BM soup-treated groups, 70.6% ± 3.2% and 71.6% ± 5.5% respectively, which were significantly higher than the deactivated BM Soup and saline control groups (*P* < 0.05, Fig. 2b). Histological observations showed that the acini structure was damaged in the saline control group and the deactivated BM Soup group, but well preserved in BM Soup group (Fig. 2c,f). Von Willebrand Factor staining showed that the number of blood vessels in saline control or deactivated BM soup-treated group was ~50% lowered than blood vessels in the sham-IR and BM soup-treated mice (*P* < 0.05, Fig. 2g). PCNA staining revealed that BM soup-treated mice had a higher percentage of proliferating cells than the deactivated BM soup group (Fig. 2h, *P* < 0.05). Subpopulations of salivary cells were detected by immunofluorescent staining. More cells were positive for Aquaporin 5 (AQP5, marker of acinar cells), alpha-smooth muscle actin (α-SMA, marker for myoepithelial cells), Keratin 5 (CK5) and c-Kit (markers for ductal and progenitor cells), and GDNF family receptor alpha-2 (GFRα2, marker for parasympathetic nerves) were observed in BM Soup-treated group when compared with the IR + SC or deactivated BM Soup treatment (Fig. 3, *P* < 0.05). The expression levels of genes related to tissue repair/regeneration were analyzed by quantitative RT-PCR (Fig. 4). All genes tested in this study were up-regulated after BM Soup treatment. Specifically, expression of AQP5 (acinar cell), CK5 (ductal cell), VEGF, NGF and Cyclin D1 (cell cycle G1/Synthesis) were significantly higher in BM soup-treated group than that of the deactivated BM soup (*P* < 0.05).

### BM Soup contained angiogenesis growth factors and cytokines that may play important role in functional SG restoration

**Figure 5:**
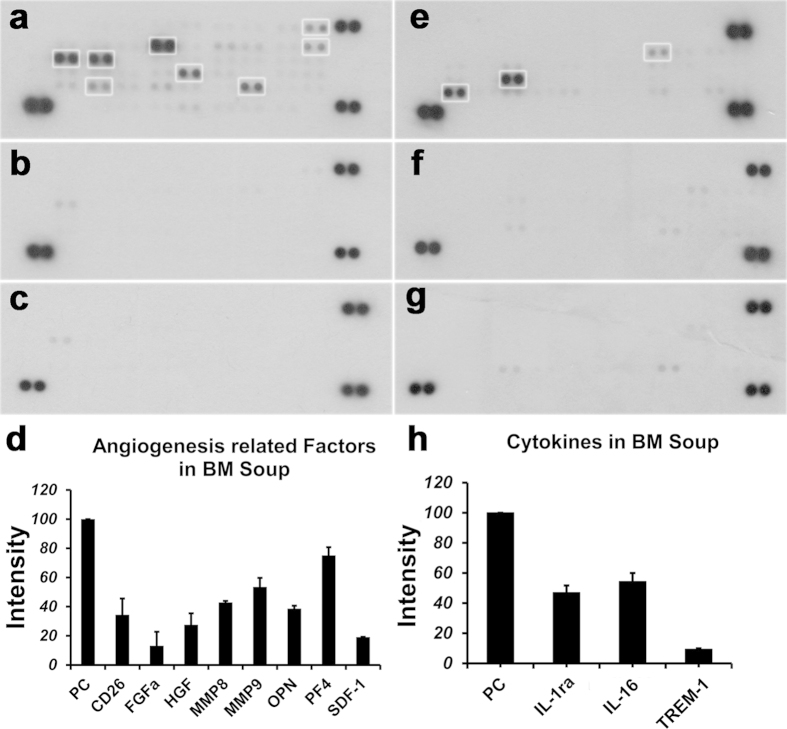
Characterization of BM Soup and its deactivated form. A protein microarray approach was used to characterize the angiogenesis-related growth factors in BM Soup (**a**) and deactivated BM Soup (**b**), while normal saline control (**c**) was used as a negative control. A cytokine microarray was also used to profile cytokines in the BM Soup (**e**), deactivated BM Soup (**f**) and normal saline control (**g**,**d**,**h**) after background subtraction and removal of overlaps in normal saline control, quantification of factors (intensity) was shown. Several angiogenesis-related proteins and two cytokines were identified in BM soup. After the deactivation process using proteinase K + 95 °C, all these factors and cytokines were below the detection level of the two protein arrays. All data were mean ± S.D. from three independent experiments. All data were normalized to the intensity of positive control (PC, three pairs of dots at the corners).

We hypothesized that functional restoration of SGs is attributed to the presence of protein factors secreted/released by bone marrow cells. Thus a protein microarray approach was used to characterize the angiogenesis-related growth factors in BM Soup. Several angiogenesis-related proteins were identified in BM soup (Fig. 5a). Specifically, factors related to tissue repair/regeneration such as MMP-8, -9, FGF-1, HGF, OPN and SDF-1, and some anti-angiogenic factors (PF4 and CD26) were found to be present in BM Soup. A cytokine microarray was also used to profile cytokines in the BM Soup. Two cytokines, IL-1ra and IL-16, were found to be at abundant levels in BM Soup (Fig. 5e). After the deactivation process using proteinase K + 95 °C, all these factors and cytokines were below the detection level of the two protein arrays used in this study (Fig. 5b,f). A negative control group using normal saline injection into IR mice was also tested on the protein arrays (Fig. 5c,g). Overall, the protein array results demonstrated that: 1) Proteinase K and heat were efficient in decreasing the proteins that were initially detected on the array for the ‘Native BM Soup’ (Fig. 5a,e), and 2) a preliminarily screen of some angiogenesis-related growth factors and cytokines in the ‘Native BM Soup’ was done but we have not confirmed yet which of these factors were responsible for the therapeutic effects. The limitations of protein arrays are enumerated in the Discussion section.

## Discussion

The findings of this study were: 1) that the protein components in the BM Soup were the active/therapeutic ingredients for functional salivary restoration following IR, and 2) that some of the protein components in BM Soup were angiogenesis-related growth factors and cytokines.

To confirm protein factors were the active ingredients, BM Soup was digested by proteinase K (“Deactivated BM Soup”) and injected into head and neck IR mice. The strategy of using proteinase K combined with heating at 95 °C served three purposes. First, this method allowed the injection of the “Deactivated BM Soup” safely into mice. Second, proteinase K cleaved active proteins into (smaller) peptides/amino acids and this would suggest that the active proteins were the therapeutic ingredients in BM Soup (and not the cleaved peptides). Third, proteinase K is commonly used in molecular biology to digest proteins and remove contamination from preparations of nucleic acid (i.e., to isolate highly native, undamaged DNAs or RNAs). Thus for nucleic acids, the BM Soup that was treated with proteinase K would still have native (or active) nucleic acids. Our hypothesis behind this study is that proteins (rather than DNA, lipid or carbohydrate) are the main molecules that execute the cellular function (intracellularly or extracellularly). While lipid and carbohydrate are stable molecules that would survive the proteinase K with heat treatment, we know that heating at 95 degrees denatures double stranded DNA. However, we do not think DNA plays an important role of stimulating development, regeneration and differentiation of the salivary gland cells. For lipids and carbohydrates, there are no reports in the literature regarding the cleavage of lipids and carbohydrates by proteinase K. Also based on our experience with biochemistry, lipids and carbohydrates should be stable at a temperature of 95 °C. There are two protease inhibitors that can totally inactivate proteinase K, which are Diisopropyl fluorophosphates (DFP) and phenylmethylsulfonyl fluoride (PMSF). DFP is much more toxic than PMSF. PMSF covalently binds to the serine amino acid present in the active site of the protease. Any remaining PMSF is rapidly degraded in water. The half-life in aqueous solutions is 110 min at pH 7, 55 min at pH 7.5, and 35 min at pH 8, all at 25 °C. However, we were concerned that both proteinase K and PMSF would still be toxic when injected into IR mice and thus demonstrated that heating with 95 °C removed the 28.9 kDa band (proteinase K) from the gel (Fig. 1b).

Although our chosen method of proteinase K with 95 °C heating seems to be simple minded, it made the point, based on our experience in bioanalytical chemistry, that proteins (and not DNA, RNA, lipids, carbohydrates) were the active ingredients in BM Soup that are responsible for the treatment. There are of course other strategies, based on our experience in bioanalytical chemistry and from recommendations through the review of this study, which could have been used. For example, if the purpose was to rule out the role of small molecules (such as lipids or carbohydrates) in the BM Soup treatment, then using ultracentrifugation and a 3 kDa cutoff filter (such as the Amicon Ultra Filter) would have contained the proteins, nucleic acids, and other large molecules (everything above 3 kDa) in the filter top while the filtrate (flow-through) would contain lipids, carbohydrates, amino acids, and small peptides. Injecting the components in the filter top would have resulted in a positive effect in irradiated SG while injections of the filtrate into IR mice would not have any treatment effect. Alternatively, we could use an approach consisting of “BM Soup + proteinase K + Amicon Ultra Filter (3K Cutoff)”. The filter top would only contain proteinase K (and some large peptides derived from the proteinase K cleavage) while the filtrate would contain lipids, carbohydrates, amino acids, peptides (including those derived from proteinase K-cleaved proteins). Both filter top and filtrate should not have any treatment effect if injected into IR mice. Additional methods to separate specific components in BM Soup could have been protein deglycosylation, isolation of lipid droplets from cells by density gradient centrifugation, or treatment with nucleases to remove nucleic acids. However with every additional method of separation, there would be a requirement for a higher number of IR mice for the *in vivo* injection experiments. Thus we selected, based on our experience with biochemistry, the use of proteinase K plus 95 °C heating as the most convenient and straightforward method to demonstrate proteins were the active ingredients in BM Soup.

Cotrim and coworkers tested the notion that injury to the adjacent microvasculature played a role in SG radiation damage by demonstrating that FGF and VEGF gene transfer protected mouse SG endothelial cells that underwent head and neck IR^24^. Results from our study also indicated a significant reduction in the number of micro-vessels in irradiated SG. Thus, we focused on identifying angiogenesis-related factors in BM Soup using a protein microarray approach. The microarray analysis indicated that matrix metallo-proteinase-8 (MMP-8), MMP-9 and osteopontin (OPN) were among the highly expressed factors in BM Soup. Their roles in angiogenesis were reported as such: MMP-8 and -9 degraded the matrix surrounding endothelial cells, and then promoted their migration and proliferation, resulting in angiogenesis^25^,^26^. OPN inhibited the apoptosis of vascular endothelial cells, promoted their migration, and induced angiogenesis of endothelial progenitor cells^27^,^28^. FGF-1 and HGF, another two growth factors expressed in BM soup, were reported to participate in the development of SG^29^,^30^. Stromal cell derived factor-1 (SDF-1) and its receptor CXCR4 play a critical role in the migration of cells and other biological process, including vascularization, immune response and neurogenesis^31^,^32^,^33^,^34^. SDF-1 expression is increased during the response to tissue injury and induces progenitor/stem cells with CXCR4 to migrate from the bone marrow to the damage site^35^. Wang and colleagues reported that the SDF-1/CXCR4 pathway was critical for MSCs to migrate and improve the functional restoration of SG in mice with Sjögren’s-like disease^36^. Moreover, local injections of SDF-1 mobilized progenitor cells from bone marrow to the circulation and enhanced vascularisation^37^. The relationship between CD26 and angiogenesis remains controversial. There are reports that CD26 inhibited the migration of endothelial cells and formation of capillaries^38^, while other reports found CD26 stimulated the proliferation and migration of endothelial cells^39^. PF4, also named as CXCL4, is a negative modulator of angiogenesis by inhibiting proliferation and migration of endothelial cells^40^. Two cytokines (moderately expressed) were also identified in the BM Soup by the cytokine microarray. IL-1 receptor antagonist (IL-1ra) is the endogenous inhibitor of IL-1. It is secreted by mononuclear cells and monocytic phagocytes^41^,^42^. IL-1ra modulates IL-1 related immune and inflammation activities by blocking the IL-1 receptor. IL-1ra was reported to stimulate macrophages into a wound healing phenotype and promoting endothelial cell migration^43^. IL-16 (a CD4-binding T cell active cytokine) is a chemoattractant for CD4 + cells^44^. IL-16 can also inhibit pro-inflammatory factors, such as Th2 cytokines, IL−4, −5 and −13^45^. It was surprising to find PF4 as the highest expressed factor in BM Soup, and still this did not negatively affect the angiogenesis process in irradiated SG. Another interesting observation from our protein array results was that some previously reported growth factors involved in SG development, repair, or regeneration such as PDGF, IGF, VEGF and other FGFs (such as FGF-2 and KGF) were not detected in BM Soup. This led us to believe that the repair of acutely irradiated SG could happen with a mixture of (growth) factors, either pro- or anti-angiogenesis, which were somehow well-orchestrated (like a cascade) during the salivary tissue repair process and this resulted in an overall improved salivary function. However, we should be warned that there are reports that injections of multipotent stem cells lead to maldifferentiation^46^ and neoplasia^47^. Theoretically, BM Soup is a cell-free therapy and should possess a lesser risk to become tumorigenic. BM Soup contains fewer histocompatibility antigens, such as MHC-I and MHC-II, than the intact cells and should theoretically elicit a weaker immune response. But, since BM Soup also contains several angiogenesis-related growth factors and cytokines (as shown in this study), additional experiments are required to investigate its effect, if any, on tumor cell growth.

Using protein arrays was a cost-effective strategy for preliminary identification of potential factor proteins in BM Soup. The limitation of the method was its relative quantification. Therefore protein arrays could not provide an exact concentration, for example, of a growth factor in BM Soup injected *in vivo* into an irradiated mouse. In future experiments, ELISA will be used to measure the actual level of each major factor in BM Soup. The second limitation of this study was that the importance of each (or a group of) identified growth factors/cytokines in restoring salivary function remained unknown. In future studies, we will use neutralizing antibodies against selected growth factor/cytokine to create a “specific factor-depleted BM Soup” that will be injected into irradiated mice to test their roles in the salivary gland restoration process. One last limitation: there were certainly additional (known and unknown) active protein factors in BM Soup that this study did not screen. Still, our strategy of using protein arrays facilitated the screening of BM Soup for factors and cytokines.

This preliminary screening of growth factors and cytokines released from BM Soup will assist future studies aiming to identify BM Soup-targeted genes and pathways that exert the functional restoration of irradiated SGs. These genes/pathways can be identified by using the gene network analysis of global expression alterations caused by BM Soup treatment. We have recently used a proteomics approach to quantify 1850 proteins, based on liquid chromatography with tandem mass spectrometry (LC-MS/MS) and tandem mass tagging (TMT), for global analysis of proteins in SG cells between BM Soup-treated and untreated NOD mice^22^. Many SG proteins in Sjögren’s-like disease mice were found to be altered by the BM Soup treatment. Our future studies will use both genomic and proteomic analysis of the SG acinar/ductal cells in BM Soup-treated irradiated mice. Gene network/pathway analysis can be based on RNA-Seq data because this high-throughput technology provides a more comprehensive coverage of the genome than proteomic analysis. However, proteomics methods (such as quantitative mass spectrometry and immunoassays) are still needed because they will allow confirmation of the RNA-Seq findings at the protein level.

This study tested the benefit of BM Soup as a mitigator to minimize IR-toxicity to SG. BM Soup was injected from 5~7 days post-IR, which was during the early effect of IR-injury to SG. We selected to inject BM Soup 5 to 7 days post-IR because, based on our experience working with irradiated mice, we have noted that both the IR and the tail vein injection procedures were stressful events for the mice. Thus mice were allowed to recuperate in the animal facility center for 5 to 7 days post-IR before the first injection of BM Soup (this was our humane approach to treat IR mice). We do not know the therapeutic effect of BM Soup, if injected prophylactically, (i.e., in situations before IR therapy such as a radioprotector), or in a situation where there was already an established reduction in salivary flow (such as at 8 weeks post-IR in mice). Thus these situations remain to be tested. This study used a single fraction dose of 15 Gy, as compared to fractionated modulated radiation therapy that patients are receiving. Still our group was able to simulate the critical clinical conditions such as: 1) the irradiation was performed in clinically realistic conditions with respect to type of radiation (high-energy 6 MV photons from a clinical Varian Clinac 6EX linear accelerator applied to the salivary gland area with sufficient dose build-up), and 2) sparing of normal tissues by applying an accurately collimated slit field with dosimetry characterized using small-field detectors^48^ and with out-of-field dose well below 3%. We are currently adapting three different fractionated IR regimens, previously reported by Baum and colleagues, for the use with the Varian Clinac 6EX linear accelerator. Within these above mentioned conditions, BM Soup mitigated IR-injury by increasing saliva secretion and by protecting several cell populations in SG. Interestingly, we observed with immuno-staining of GFRα2 (used here as a marker for parasympathetic nerves) that BM Soup preserved the parasympathetic innervations while the deactivated BM Soup did not. It has been documented that parasympathetic nerve played a crucial role in SG secretion and development^49^,^50^. After parasympathectomy, gland regeneration was impaired^50^. Moreover, Hai and colleagues reported that improved parasympathetic innervations restored SG function damaged by IR^51^. Our hypotheses are that: 1) some components of BM Soup protected directly the parasympathetic neurons from IR-damage, or 2) that BM Soup treatment rescued acinar, myoepithelial and endothelial cells in the SG (Fig. 3a). These cells in turn secreted neurotropic factors, such as NRTN^52^ and GDNF^53^, which protected parasympathetic innervations from injury and promoted tissue regeneration^50^. Further investigations will be required to define these underlying mechanisms.

## Materials and Methods

### Preparation of BM Soup

BM Soup was prepared as described previously^21^. Briefly, each set of experiments was performed using bone marrow pooled from five 8-week old male C3H (donor) mice. BM cells were flushed from tibias and femurs in sterile PBS. The cell suspension was filtered through a 40 μm cell strainer, followed by centrifugation at 1,500 rpm for 5 min. The cell pellet was resuspended with normal saline to a concentration of 10^7^ cells/100 μl. The suspension was processed by three cycles of freeze-thaw with dry ice and a 37 °C water bath. Insoluble materials were removed by micro-centrifugation at 13,500 rpm for 30 min at 4 °C. The supernatant (BM Soup) was kept on ice before injections into the tail vein of recipient C3H female mice. For experiments used in this study, the BM Soup was obtained from two sets of five pooled donor mice and had a protein concentration of ~1.5 μg/μl.

### Deactivation of BM Soup

Initially, four methods to deactivate BM Soup were tested. 1) Heated at 95 °C for 20 min. The micro-centrifuge tube cap was wrapped with parafilm to avoid evaporation; 2) Heated at 95 °C for 60 min; 3) Digested by Trypsin (1:20 w/w, T1426, Sigma-Aldrich, ST. Louis, USA) overnight at 37 °C; and 4) Digested by 0.1 μg/μl proteinase K (P2308 Sigma-Aldrich, ST. Louis, USA) overnight at 37 °C. Precipitates were removed by micro-centrifugation by 13,500 rpm for 15 min. 12% sodium dodecyl sulfate-polyacrylamide gel electrophoresis (SDS-PAGE, 120V, 60 min) and Coomassie blue staining were conducted to test the efficiency of deactivation. The total protein concentration was measured by the bicinchoninic acid assay method (23225, BCA; Thermo Scientific, Pierce, IL, USA). Proteinase K digestion was the most efficient method. To safely inject into animals, 95 °C heating for 20 min followed by 13,500 rpm micro-centrifugation for 15 min was performed to remove/inactivate proteinase K. The supernatant, named “deactivated BM Soup”, was kept on ice before injections into the tail vein of recipient C3H female mice.

### Characterization of BM Soup and its deactivated form

The angiogenesis-related factors and cytokines within the native BM Soup and deactivated BM Soup were profiled by Proteome Profile Mouse Arrays (ARY015, ARY006, R&D Systems, Minneapolis, USA). All procedures were according to the manufacturer’s instructions. Briefly, 200 μl of BM Soup, deactivated BM Soup, or normal saline was mixed with a cocktail of biotinylated detection antibodies, and incubated overnight with Proteome Profile Mouse Array. The membranes were washed to remove the unbounded materials. Streptavidin-HRP and chemiluminescent detection reagents were used, followed by exposing membranes to X-Ray films. After subtracting background and removing the overlaps in normal saline control, the densities of signals on membranes corresponding to the amount of proteins were analyzed by Image J software (NIH).

### Animals

Female C3H mice of eight weeks old purchased from Charles River (Montreal, QC, Canada) were used as recipient mice, while age-matched male C3H mice were donor mice. The mice were kept under clean condition with free food and water in animal resource center at McGill University. All experimental procedures were performed in accordance with guidelines imposed by the Canadian Council on Animal Care and approved by the University Animal Care Committee (UACC) at the McGill University (Approved protocol #5330, www.animalcare.mcgill.ca).

### Irradiation (IR)

Eight week-old female C3H mice were anesthetized with 0.3 μl/g body weight of 60 mg/ml Ketamine and 8 mg/ml Xylazine (02239093, Novopharm, Toronto, Canada) cocktail given by intra-muscular injection, and restrained in a container for irradiation. Salivary glands were damaged by exposing them to a single 15Gy of radiation from a Varian Clinic 6EX linear accelerator (6 MV, build-up provided). The radiation was collimated to the head and neck area to guarantee less than 3% beam strength in the rest of body. After recovering from anesthesia, mice were returned to their cage and maintained in animal facility for 8 weeks post irradiation. 100 μl vehicle (normal saline), native or deactivated BM Soup was injected at 5~7 days post-irradiation, twice a week for two consecutive weeks, through the tail vein. Twenty mice were divided into 4 groups (five mice in each group): (1) Sham irradiation group (no irradiation and no injection); (2) Irradiation with injection of normal saline; (3) Irradiation with injection of BM Soup; (4) Irradiation with injection of deactivated BM Soup.

### Salivary flow rate (SFR) measurement

Female mice were weighed and anaesthetized by intra-muscular injection of 0.3 μl/g body weight of a 60 mg/ml ketamine and 8mg/ml xylazine mixture. Saliva secretion was stimulated by injecting 0.5 mg/kg body weight of Pilocarpine (P6503, Sigma-Aldrich, ST. Louis, USA) subcutaneously. Whole saliva was collected into a 0.5 ml microcentrifuge tube for 10 min. The saliva volume was determined gravimetrically, assuming its density is 1 g/ml. Salivary flow rate (SFR) was determined by volume of saliva/10 min/g body weight at pre-irradiation, week 4 post-IR and week 8 post-IR. At time of sacrifice (week 8 post-IR), the mice submandibular glands were harvested.

### Histology staining

#### H&E staining

As previously described, half of a submandibular gland was fixed in 4% PFA and embedded in paraffin. Tissue sections were cut at 5–8 μm thickness and stained by Hematoxyline and Eosin (H&E). The percentage of surface area occupied by acinar cells/total area was calculated by two independent examiners under 200X magnification of 10 fields per gland/mouse with NIH image J software

#### PCNA staining

Cell proliferation staining was performed with the Zymed PCNA staining kit (931143, Invitrogen, Carlsbad, CA, USA). After deparaffinization and rehydration, five-micrometer thickness sections were blocked in 10% H_2_O_2_ in Methanol for 10 min. Before primary antibody labeling, tissue sections were treated three times with 10 mM Citrate Buffer solution (pH 6.1) in a 600 W microwave for 5 min and then cooled down to room temperature for 30 min. Thereafter, slides were preceded with IHC routine with manufacturer’s instruction. Two examiners independently counted the PCNA positive cells percentage in a blind manner under 400X magnification of 10 fields per gland/mouse with Image J software (NIH).

#### Blood vessel/capillary density

Five-micrometer thickness tissue sections were stained with the antibodies to von Willebrand Factor (vWF, ECM595, Millipore Corporstion, Billerica, MA01821, USA) following the manufacturer’s instruction. Briefly, after deparaffinization and rehydration, antigen retrieval was done (mentioned above). Tissue slides were incubated with rabbit anti-mouse vWF for 2 hours at room temperature. Then Goat anti-rabbit secondary antibody was used for 15 min at room temperature. The number of blood vessels was counted under 200 X magnification of 10 fields/gland with Image J software (NIH).

#### Immunofluorescent staining

Frozen submandibular gland sections (6–8 μm) obtained from C3H mice were fixed in 4% PFA (P6148, Sigma-Aldrich, ST. Louis, USA) for 10 min, and then blocked in 10% normal donkey serum for 1 hour to inhibit the endogenous biotin activity. These antibodies were used: (a) goat anti-aquaporin 5 (1:50, AQP5, G-19, sc-9890, Santa Cruz Biotech, Santa Cruz, CA, USA); (b) rabbit anti-alpha smooth muscle actin (1:100, α-SMA, ab7817, Abcam, Cambridge, MA, USA); (c) rabbit anti-cytokeratin 5 (1:500, CK5, Sigma-Aldrich, Oakville, ON, Canada); (d) rabbit anti-c-Kit (1:100, ab5506, Abcam, Cambridge, MA, USA); (e) goat anti GFRα-2 (1:100, R&D systems, Minneapolis, USA); Negative controls without primary antibodies were used. Sections were incubated with primary antibodies (or PBS) at 4 °C overnight. After 3 times washing in PBS, slides were incubated with secondary antibodies (1:100) in the dark for 1 hour at room temperature. Secondary antibodies were donkey anti-goat- fluorescein isothiocyanate-conjugated (FITC), anti-rabbit-FITC, anti-rabbit-Alexa Fluor®594-conjugated, or anti-goat-Alexa Fluor® 594-conjugated. Then, 4,6-diamidino-2-phenylindole, dihydrochloride (DAPI, Invitrogen, San Francisco, CA, USA) was added for 1 min to label the nucleus of cells. Fluorescence pictures were taken by Leica DM4000 fluorescent microscope. The intensity of fluorescence signal was calculated with Image J software (NIH) for at least 5 fields of 200X per gland/mouse.

#### Quantitative real-time PCR (qRT-PCR)

Total RNA was extracted from the mouse submandibular gland tissue with TRIZOL reagent (15596018, Invitrogen, Carlsbad, CA). 25 ng RNA per sample was used for first strand cDNA synthesis with Thermoscript RT-PCR system (11146-016, Invitrogen, Carlsbad, CA). Triplicate qRT-PCR assays were performed by Step One Plus (Life Technologies) in TaqMan Universal Master Mix II (4440040, Applied Biosystem, Foster City, Canada). The probes used were for HGF (assay ID:Mm01135193), EGF (assay ID: Mm00438696), NGF (assay ID: Mm00443039), Cyclin D1 (assay ID: Mm00432359), BMP7 (assay ID: Mm00432102), AQP5 (assay ID: Mm00437578), IGF-1R (assay ID: Mm00802841), FGF2 (assay ID: Mm00433287), MMP2 (assay ID: Mm00439498), CK5 (assay ID: Mm00503549), VEGF (assay ID: Mm01281449), GFRα2 (assay ID: Mm00433584) and TGFb1 (assay ID: Mm01178820). The expression levels of 16 endogenous control genes in mouse salivary gland tissue were first tested by TaqMan^®^ Array Mouse Endogenous Control Plate (4426701, Life Technologies, USA). The results demonstrated that three genes, GAPDH, β-ACTN and 18sRNA, were stably expressed and unaffected by irradiation and BM Soup/De-BM Soup treatment. Thus we selected Glyceraldehyde-3-phosphate dehydrogenase (GAPDH, assay ID: Mm99999915) as the endogenous control gene to normalize for variations in sample input for relative quantification of gene expression in qRT-PCR experiments. Three experimental replicates were conducted for each sample. PCR was run at 50 °C for 2 min, 95 °C for 10 min, and 40 cycles [95 °C for 15 s, 60 °C for 1 min].

#### Statistical analysis

SPSS version 19 software was used to perform the statistical analysis. All data are presented with mean ± S.D. and were analyzed by Student’s *t*-test or one-way ANOVA with Turkey’s Post-Hoc. The statistical significance was defined as *P* < 0.05.

## Additional Information

**How to cite this article**: Fang, D. *et al.* Identification of the active components in Bone Marrow Soup: a mitigator against irradiation-injury to salivary glands. *Sci. Rep.* **5**, 16017; doi: 10.1038/srep16017 (2015).
